# Supplementary material for: Hippocampus segmentation on epilepsy and Alzheimer's disease studies with multiple convolutional neural networks
Source: Heliyon. 2021 Feb 10;7(2):e06226. doi: 10.1016/j.heliyon.2021.e06226 (PMC7892928; doi:10.1016/j.heliyon.2021.e06226)
Supplement: MMC — This appendix presents some experiments related to optimizing our methodology as a whole, including choice of optimizer, loss functions, and the consensus approach. [file mmc1.pdf]

Appendix

Training

This appendix presents some experiments related to optimizing our methodology as a whole, including choice of optimizer, loss functions, and the consensus approach.

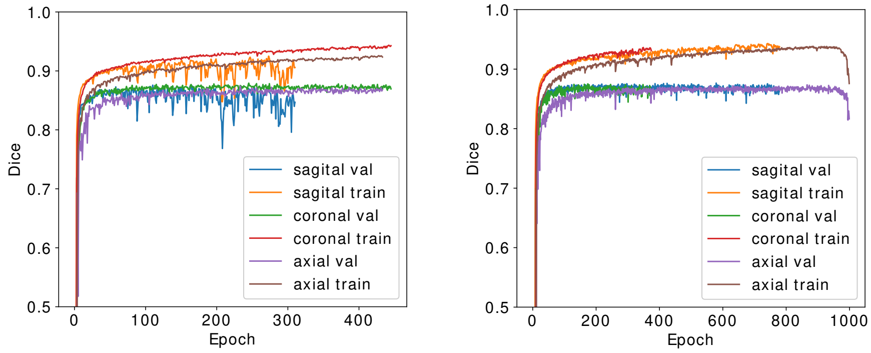

(a) (b)

Figure S. 1: Validation and training Dice for all models, using: (a) ADAM (b) RADAM. Both with same hyperparameters and no stepping. Early stopping is due to patience. RADAM displays more stability. Although overfit is noticeable in (b), training was stopped by patience and only the best validation weights are used.

Optimizers, Learning Rate and Scheduling

Training hyperparameters are the same for all three networks. Regarding the optimizer of choice and initial LR, grid search defined 0.0001 with ADAM [1] and 0.005 LR with SGD [2] to deliver similar performance. The recent RADAM from Liu et al. [3] with 0.001 initial LR ended up being the optimizer of choice, due to improved training stability and results (Fig S. 1). LR reduction scheduling is used, with multiplication by 0.1 after 250 epochs, its impact is showcased on Figure S. 2(a). While training on HarP with an 80% holdout training set, an epoch consisted of going through around 5000 sagittal, 4000 coronal and

3000 axial random patches extracted from slices with presence of hippocampus, depending on which network is being trained, with a batch size of 200. The max number of Epochs allowed is 1000, with a patience early stopping of no validation improvement of 200 epochs. Note that weights are only saved for the best validation Dice.

Hyperparameter Experiments

Some of the most important hyperparameter experiments can be seen in Table S. 1. These showcase the impact of Boundary Loss and RAdam in relation to more traditional approaches. Results from each change in methodology were calculated using the full consensus and post-processing. For these experiments, holdout of 80/20% on HarP was used, keeping Alzheimer's labels balanced. Reported Dice is the mean over the 20% test set.

**Optimizer**	**LR**	**Loss**	**HarP (Dice)**
SGD	0.005	Dice Loss	0.8748
ADAM	0.0001	Dice Loss	0.8809
ADAM	0.0001	GDL	0.8862
ADAM	0.0001	Boundary	0.9068
RADAM	0.0001	Boundary	0.9071
RADAM	0.001	Boundary	**0.9133**

Table S. 1: Some of the most relevant hyperparameters experiments test results, in a hold-out approach to HarP. The bolded result represents the final model. All tests in this table use 64^2 patch size and the modified U-Net architecture.

Early experiments showed that for the patch selection strategy, 80/20% provided the best balance between positive and negative patches, with 64^2 patch size. Implementation of Boundary Loss resulted in slightly better test Dice than Dice Loss. We found that augmentation techniques only impacted Dice results in HarP slightly, sometimes even making results worse. Augmentation's most relevant impact, however, was avoiding overfitting and very early stopping due to no validation improvements in some cases, leading to unstable networks.

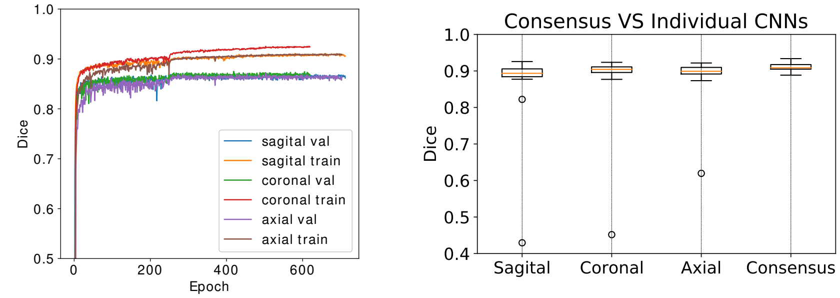

(a) (b)

Figure S. 2: (a) Training and validation Dice curve for the best model, with RADAM and LR step. (b) Boxplot for HarP test models, showing the improvement in variance and mean Dice from the Consensus compared to using only one network.

We found that, as empirically expected, the consensus of the results from the three networks brings less variance to the final Dice as seen in Figure S. 2(b), where the result of isolated networks are evaluated in comparison to the consensus. Early studies confirmed that 0.5 is a reasonable value to choose for threshold after the activation averaging. Attempts at using a fourth 3D UNet as a consensus generator/error correction phase did not change results significantly. Since the best performing network varied according to hyperparameters, we choose to keep a simple average of activations instead of giving more weight to one of the networks.

References

[1] D. P. Kingma, J. Ba, Adam: A method for stochastic optimization, arXiv preprint arXiv:1412.6980.

[2] Y. Bengio, I. J. Goodfellow, A. Courville, Deep learning, Nature 521 (2015) 436–444.

[3] L. Liu, H. Jiang, P. He, W. Chen, X. Liu, J. Gao, J. Han, On the variance of the adaptive learning rate and beyond, arXiv preprint arXiv:1908.03265.
